# Using infrared cameras in drones to detect bleeding events

**DOI:** 10.1186/s12873-023-00912-9

**Published:** 2023-12-01

**Authors:** Christoph West, Bernhard Kaus, Sean O’ Sullivan, Henning Schneider, Oskar Seifert

**Affiliations:** 1 University of Applied Sciences Giessen, Wiesenstrasse 14, 35390 Giessen, Germany; 2 Justus-Liebig-University Giessen, Ludwigstrasse 23, 35390 Giessen, Germany

**Keywords:** Drone, Hemorrhage, Mass casualty incident, Thermal imaging

## Abstract

**Background:**

Hemorrhage is one of the main causes of death in trauma. Critical bleeding in patients needs to be detected as soon as possible to save the patient. Drones are gaining increasing importance in emergency services and can support rescue forces in accident scenarios such as a mass casualty incident.

**Methods:**

In this study, a simulated pelvic hemorrhage was detected using a drone from 7 m above the ground over a time span of 30 s.

**Results:**

The results allow a good detection of the pelvic hemorrhage. Nevertheless, the simulated blood cools down quickly. After 30 s, there was no significant temperature difference compared to the rest of the body. At this point, further assessment is only possible via the RGB image.

**Conclusion:**

The findings suggest that bleeding from an open and continuously bleeding wound would most likely be detectable using the drone’s thermal imaging camera, even over a longer period of time.

## Background

Hemorrhage is one of the main causes of death in trauma. If the source of traumatic blood loss is not controlled within the first hours, the risk of death is substantially increased [1–3]. In 30% of severely injured patients, critical bleeding is the main cause of death. Furthermore, unrecognized or stopped hemorrhage is one of the most common causes of preventable death in severely injured patients [4, 5]. Therefore, death because of critical bleeding is a major and relevant part of prehospital emergency medical treatment and must be recognized as early as possible [3, 6].

Meanwhile, many clinically invasive approaches, such as prehospital thoracotomy [7, 8] devices, such as the REBOA system [9, 10], have been developed for early intervention in a preclinical environment and have been adapted in many regions. Additionally, public awareness campaigns such as “Stop the Bleed” [11] have been introduced to train civilians in early hemorrhage control. However, before such treatment options can be applied, patients need to be recognized and accessed by emergency response teams – for example, in mass casualty incidents.

Especially in unclear or developing emergency scenarios that can differ in size and length or involve many different casualties and present with a variety of severities in injuries, early recognition of patients, especially those with hemorrhage, is even more relevant [12]. In regards to the location or geography, emergency scenarios can also be challenging because of forests, differences in heights (mountains, bridges, hills etc.) or even light conditions (day/night/evening) which can hinder access to the incident site. Furthermore, weather conditions such as rain and cold weather can reduce the body temperature of exposed patients.

As these circumstances can lead to a reduction in body temperature, the complex biochemical reactions that are performed to sustain a level of homeostasis in humans can therefore be put out of balance [13]. For example, a reduced body temperature was shown to be associated with reduced enzyme activity. Especially in regard to homeostasis, the function to stop and prevent bleeding, coagulation and platelet functions are limited [14, 15].

Furthermore, hypothermia combined with coagulation and acidosis especially in trauma patients, is also known as the lethal triad, which consists of hypothermia, acidosis, and coagulopathy and is associated with increased mortality and morbidity [13, 16, 17].

The use of drones in rescue services and fire departments has become increasingly important in recent years. With the help of drones, areas with accidents can be flown over quickly to create a picture of the situation. Drones can also act as cost-effective alternatives to a helicopter when searching for people. Helicopter crews do not have to be put at risk.

This work will investigate whether drones equipped with a thermal imaging camera are able to identify bleeding in an accident victim as such. If the extent of the bleeding can be identified from the air, adequate and improved patient care can be ensured.

To find a link to the study structure and the experiments, we give a brief introduction to the chemical and physical properties of heat and the functionality of thermal imaging cameras. The phenomenon of heat, also referred to as infrared radiation (IR), is fundamentally the oscillation of atomic particles. The thermal signature emitted by objects is a direct result of the degree of motion exhibited by their constituent atoms - a higher degree of motion corresponds to elevated temperatures. The study and application of these thermal signatures, known as thermography, allows for the practical utilization of thermal imagery.

Although humans possess the ability to perceive heat, the visualization of infrared radiation remains elusive due to its occurrence at an electromagnetic wavelength imperceptible to the human eye. To render these infrared signatures observable, thermal cameras have been engineered to convert them into a format discernible by humans.

Thermal cameras employ specialized lenses capable of detecting infrared frequencies, in conjunction with thermal sensors and image processors, to present the results on a visual display. When affixed to a drone, such cameras are typically mounted on a gimbal, ensuring image stabilization and facilitating a full 360-degree rotation of the lens. The thermal sensors incorporated in these sophisticated cameras, formally referred to as microbolometers, have undergone significant advancements in recent years. Contemporary models no longer necessitate the use of exotic cooling materials, rendering them more cost-effective.

Drone-mounted thermal cameras can ascertain the surface temperature of numerous objects, with certain exceptions. Highly polished, reflective objects exhibiting low emissivity, such as shiny surfaces, do not absorb substantial heat and are thus difficult to detect with thermal cameras. In contrast, high emissivity objects such as wood, concrete, and human beings are readily scannable. Upon capturing thermal data via their infrared cameras, drones display the information as a conventional image for operators to analyze.

Utilizing thermal imaging software, users can modify the color palette employed to represent heat within the scene. Various views can reveal distinct details within the image, with options encompassing:

White Hot: Hotter objects appear lighter, while colder areas are darker.

Black Hot: The inverse of White Hot, with hotter objects appearing darker.

Rainbow: Temperatures correspond to hues, with warm colors signifying heat.

These three options merely scratch the surface, as more advanced thermal cameras offer an expanded array of views.

The specific thermal camera employed also dictates the format in which imagery is captured and stored. While lower-end models capture images as simple files, advanced solutions incorporate thermographic data, temperature readings, and GPS tags. The DJI Thermal Analysis Tool enables further inspection of these thermographic data [18].

## Methods

In this study, hemorrhage was simulated in an injured patient and observed from the air using a drone with a thermal imaging camera.

The research recordings with data acquisition took place in the following environment. The location was part of the gliding airfield Giessen-Wieseck at coordinates: 50°36’21.00’’N; 8°43’40.24’’E. The atmospheric conditions were as follows: absence of solar heat radiation with overcast conditions (8/8) at a temperature of 2° C measured 2 m above the ground. The ground temperature on the meadow was 0.7 °C, measured with a calibrated 885 infrared camera. The wind came from the northeast at a velocity of 16 km/h with a relative humidity of 77% by a barometric pressure measuring 1038 hPa. The humidity corresponds to the average for this time of year in central Europe. On Tuesday, January 24, 2023, 2:00 p.m. − 3:00 p.m. Central European Winter Time, there was a high-pressure area. The ground on which the patient was located consisted of damp grass. Visual recordings and images of the infrared portion of the electromagnetic light spectrum were taken from a height of 7 m above the patient’s position.

### Preparation

The drone, a DJI Matrice 300 RTK and its accompanying accessories with 2x flight batteries, remote control + batteries, camera gimbal with Zenmuse H20T + memory SD-card were updated to the latest software version available at that time (02/12/2022). The batteries were fully charged, and all components were appropriately packed. The drone operator conducted a prior, thorough flight planning/preparation, incorporating pre-flight checklists. Two liters of tap water was heated to 47 °C and mixed with a packet of baking gelatin in a thermos flask, resulting in a viscous liquid resembling blood. The color of the test liquid was clear and did not play a role in these experiments.

### Setup

The test subject, a Caucasian male hereafter referred to as the patient, was in good health during the experimental procedure, free of cold/fever symptoms, and had a body temperature of 36.8 °C, a height of 191 cm, and a weight of 76 kg. These data yielded an estimated body surface area of approximately 2.04 m² using the Dubois formula [19, 20]. We assumed a front-facing body surface area of approximately 1 m². The patient was dressed in light-colored jeans and a thin, black cotton long-sleeved shirt (see RGB color recordings). After setting up and performing the preflight check of the drone, including the camera, the drone operator piloted the UAS (unmanned aerial system, also drone; in this case, a DJI M300 RTK) above the experimental site at an altitude of 7 m over the patient. The distance between the camera lens and the patient was verified prior to each new measurement series using the integrated laser rangefinder with Laser class: 1 M (according to IEC/EN 60825-1:2014), wavelength: 905 nm, measuring range: 3-1200 m (to a vertical surface with ≥ 12 m diameter and 20% reflectance), measurement accuracy: ± (0.2 m + D × 0.15%) D is the distance to a vertical surface] and corrected if necessary through manual flight inputs. The camera employed in these experiments, a Zenmuse H20T, is specifically designed for DJI drones, off-the-shelf technology and features the following capabilities: gimbal stabilization, zoom camera, wide-angle camera, thermal imaging camera, laser rangefinder, 23x optical hybrid zoom, 200x maximum zoom, high-resolution grid photos, night scene, storage: MicroSD card (max. capacity: 128 GB, UHS-1 speed class U3 required). The radiometric, thermal imaging camera has an uncooled VOx microbolometer as a sensor. The lens used has an opening angle of 40.6° with a focal length of 13.5 mm. The image resolution is 640 × 512 px with a pixel pitch of 12 μm. The camera has a thermal sensitivity of ≤ 50 mk at f/1.0 with a recording bandwidth of -40 to 150 °C at high gain and − 40 to 550 °C at low gain. Our camera recordings were always within a range of 0 to 50 °C. The specifications are provided by the manufacturer in accordance with the User Manual v1.2 from 08/2020. The assistant prepared for their role in promptly pouring the water-gelatin mixture from the thermos flasks onto the patient. Both the assistant and the drone operator were to ensure that no one except the patient would be visible within the camera frame. Simultaneously, the time between pouring the hot liquid onto the patient and initiating the recording was to be minimized, as the water-gelatin mixture’s temperature would decrease depending on the ambient/ground/patient surface temperature and the wind acting on the patient.

### Execution

**Fig. 1:**
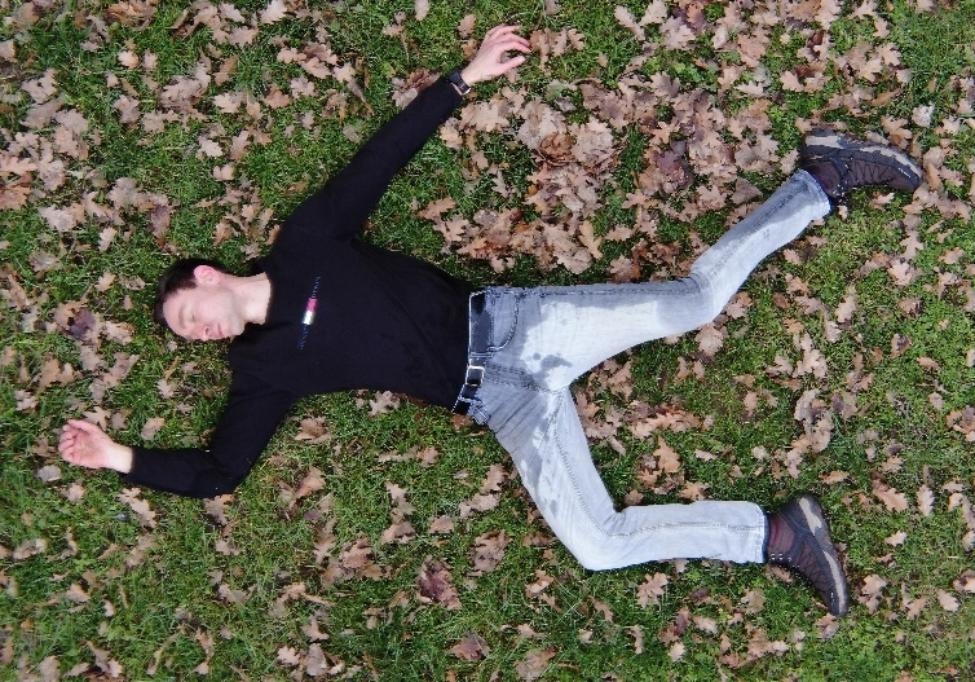
RBG image of the patient laying on the ground with blood analog on his hip area

The experiments commenced at 14:00 Central European Winter Time, with the patient lying down on a designated area of the gliding airfield’s grassland, as depicted in Fig. 1.

Subsequently, the assistant poured 200 mL to 500 mL of warm liquid over the patient, depending on the simulated bleeding source/injury. To verify that the right temperature of 37 °C was reached, an assistant used a thermometer and constantly checked the temperature of the warm liquid.

The time elapsed between pouring the liquid onto the test subject and triggering the first image was ten seconds. Two more images were taken after 7 s each. Upon capturing all scenarios, the evaluation of the images, along with their accompanying meta-information, was conducted on a computer using the DJI Thermal Analysis Tool 2 in version 2.1.8. In this tool, adjustments were made to the color gradient, representing areas with temperatures exceeding 26 °C as red. This enables a more accurate comprehension of the simulated hemorrhages.

## Results

**Fig. 2:**
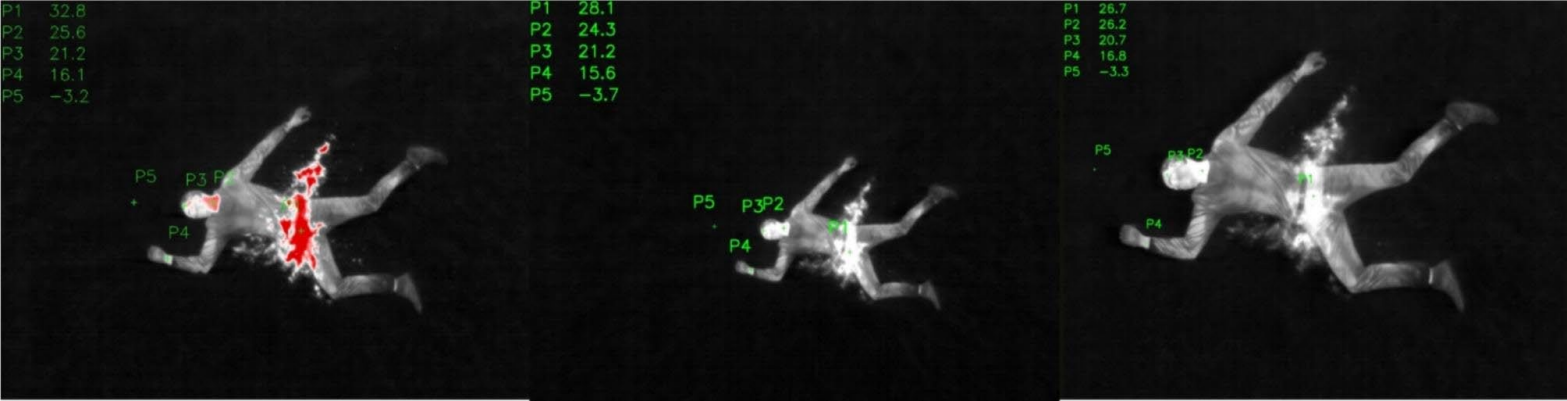
Image series with simulated, bleeding hip hemorrhage. Hip with different temperature points P1 – P5 in t_left_=10s, t_middle_=17s, t_right_=24s after pouring

The images in Fig. 2 show a clear reduction in temperature over time in the liquid representing blood. The temperature drops from initially P1 = 32.8 °C in t_left_=10 s after pouring to P1 = 28.1 °C in t_middle_=17 s after pouring (average of 0,7 K/s). After the initial drop, the chilling seems to slow down, and after an additional seven seconds, it still shows a comparable result to the third measurement at P1 = 26.7 °C in t_right_=24s after pouring (average of 0.2 K/s). After 24 s the temperature difference to the body’s surface temperature, which was measured at the neck/throat at point P2, became minimal, and the liquid would therefore become difficult to spot based on the temperature difference alone. P3 shows the temperature on the patient’s forehead, which is stable in t_left_ and t_middle_. The temperature in P3 t_right_=24s after pouring differs from those in t_left_ and t_middle_ by only 0.5 K. This deviation can already occur due to a slight lateral displacement of the head and/or incorrect setting of the measuring point within the image evaluation software and has no significant effect on to the outcome of the study. P4 represents the temperature on the inside of the patient’s right wrist. There is also a minimal, relative temperature deviation of 0.5 K here, which could be due to the reasons mentioned above. Measuring point P5 is a freely chosen reference point approximately 50 cm outside the patient in a cranial extension to the longitudinal axis of the body on the meadow. The significance of the recordings led to an absolute temperature change in the used software DJI Analysis Tool (in °C) due to the image adjustment but no change in the temperature relationships (in °K) among themselves. Thus, despite the changed measured values, an exact evaluation and a comparison of the respective situation with one another is possible.

## Discussion

The results show that recognizing blood loss in bleeding patients by using drones with infrared cameras with the help of temperature (signal strength in the picture) and characteristic geometric expansion of the liquid flow is feasible. According to Kirchhoff’s law of thermal radiation, for every body and at every wavelength, there is a proportional relationship between the absorptivity and emissivity for thermal radiation [21]. A black body (also referred to as a blackbody) is a hypothetical object that completely absorbs incident radiation of any wavelength and intensity [22]. Due to its maximum absorptivity at every wavelength, a black body also exhibits the highest possible emissivity for all wavelengths. No real body can emit more thermal radiation at a given wavelength than a black body, which thus serves as an ideal thermal radiation source [23]. Since the spectrum of the black body (also called the blackbody spectrum or Planck spectrum) depends solely on temperature, it serves as a useful reference model for numerous applications [22, 24].

The arithmetic mean of the points P5 = -3.4 °C was chosen as the reference measured value for the relative temperature measurement on the respective surfaces, since this was relatively stable over the entire period of the test series at 0.7 °C. It showed only minor relative temperature fluctuations of -0.1 to -0.5 °K, possibly due to the weather. The real measurement points P1 - P5 in the series of images therefore correspond to a relative temperature difference of + 4.1 °K.

The use of drones to detect bleeding could be beneficial for several operational scenarios, e.g., rescue services, mass casualty incidents and nighttime operations in general. In addition, it can be assumed that thermal images have an advantage over RGB images since red clothing, for example, can be better differentiated from blood.

This preliminary study has limitations. It has not yet been observed how well blood loss detection works at larger heights, above 10 m GND up to 40 m GND. The controller display resolution, the transmission bandwidth and thus the signal density are limited, and at high altitudes, the observer might not be able to distinguish between the thermal signature of the patient or possible blood loss by the resolution of the infrared camera with its lenses, which are attached to the drone. In addition, bad weather conditions in the form of fog or rain might impair the detection of blood loss at higher altitudes. Fog causes a gray/white haze in the picture, which leads to a worse determination of objects. Rain can further dilute the blood stains and cause a faster cooling of the blood outside of the body that needs to be detected.

Altough the temperature of the escaping blood drops very quickly, bleeding can still be easily detected by drone. A combination of a thermal imaging camera and RGB camera, which can also be displayed simultaneously side by side for the pilot, might improve the detection of bleeding for the pilot or search crew – even after longer periods of time.

Depending on the location of the source of the bleeding on the body and weather, minor bleeding from the palm of the patient’s hand with a low exit volume of approx. 20 mL is very difficult or impossible to detect. A direct view of what is happening from the camera drone is a key factor in successfully detecting bleeding from a patient. The flight altitude above the ground/above the patient, the resolution and the angle of aperture of the infrared camera, the ambient temperature (winter/summer), obstructed visibility due to precipitation of any kind, even dense and therefore cool fog, warm clouds of smoke, e.g., from a nearby fire or trees/leaves also play an important role and can complicate the detection of bleeding sources on the patient’s body and thus a possible remote triage via UAS. On the other hand, it is impossible to detect sources of bleeding with an infrared camera if the patient is covered by any kind of material since the camera always only picks up the surface temperature and displays it on the corresponding transmission image. This is especially the case when the body is covered by lots of thick clothing, snow/avalanches, under water, fauna and flora, other people, buildings, or is behind a pane of glass. In some cases a remote triage could be made possible with the help of merged RGB-IR night vision images in one output, since all the advantages of the respective technology or all image information are available in the respective type for the evaluator. With the help of AI-assisted image/shape recognition algorithms, which were previously trained for bleeding, a further benefit for the medical evaluator could possibly be generated. However, this completely new form of triage from the air or from a distance requires further studies.

## Conclusion

Based on the results of this study, the authors recommend the use of drones for triage of bleeding. Depending on the camera model, filter settings, resolution and the corresponding weather conditions with little or no solar radiation and precipitation, a good differentiation between the human being and the leaked blood is possible.

Further studies are necessary to investigate the applicability of blood detection by drones with infrared cameras. Standardized study designs should be used for this, whereby the following should be observed:

- Series of images with recordings of simulated bleeding with regular time intervals between the pouring out of the liquid and the triggering of the images, with the weather conditions remaining as constant as possible.
- Studies under different weather conditions during the day and night.

## Data Availability

The datasets used and analyzed during the current study are available from the corresponding author on reasonable request.

## Abbreviations

IR: Infrared radiation
UAS: Unmanned aerial system
RBG: Red green blue
